# Reasons for Living and Coping with Suicidal Ideation among Adolescents in Malaysia

**DOI:** 10.21315/mjms2018.25.5.13

**Published:** 2018-10-30

**Authors:** Normah Che Din, Norhayati Ibrahim, Noh Amit, Nor Ba’yah Abdul Kadir, Mohd Radzi Tarmizi A Halim

**Affiliations:** 1 Health Psychology Programme, Faculty of Health Sciences, Universiti Kebangsaan Malaysia, Jalan Raja Muda A Aziz, 50300 Kuala Lumpur, Malaysia; 2 Centre of Human and Societal Well-being, Faculty of Social Sciences and Humanities, Universiti Kebangsaan Malaysia, 43600 Bangi, Selangor, Malaysia

**Keywords:** suicidal ideation, adolescents, Malaysia, optimism, coping skills

## Abstract

**Introduction:**

The rate of suicide ideation everywhere continues to increase, and adolescents are therefore at risk of displaying suicidal behaviour. This study examined the protective role of the reasons for living and coping strategies in reducing suicidal ideation among young adolescents in Malaysia.

**Methods:**

A cross-sectional survey was conducted among 176 adolescents aged between 13 and 19 years of age with the majority being Malay and Muslim. The Brief Reasons for Living for Adolescents (BRFL-A), Jalowiec Coping Scale and Suicide Ideation Scale were employed.

**Results:**

The results showed that the reasons for living and palliative coping strategy correlated negatively with suicide ideation; although, further analysis using multiple regression revealed that family alliance and optimistic and palliative coping strategies were found to be significant reasons for living that protect adolescents from suicidal thoughts. Also, those adolescents who used emotive and evasive coping strategies had higher suicidal ideation.

**Conclusion:**

Cultural and social values continue to play an important role in protecting adolescents in Malaysia from suicidal behaviour.

## Introduction

While suicide is a very sensitive and stigmatised topic to discuss, preventive measures and early diagnosis of suicidal thoughts and behaviours can aid in reducing the number of deaths caused by suicide, especially in young adolescents. Even though researchers have attempted to examine and explain the reasons why people engage in suicidal tendencies and behaviours, there is no single causing factor. Rather, there are a host of factors working together that can best explain suicide. The Centre for Disease Control and Prevention (CDC) (1) ranked suicide as the third leading cause of death among adolescents aged between 15 and 19 years of age. Research conducted by Nock et al. (2) revealed that 9.2% of adolescents experience suicidal ideation, 3.1% of adolescents plan to commit suicide, and 2.7% attempted suicide, indicating that adolescents have high suicidal ideation of which the severity varies.

Reynolds (3) defined suicidal ideation as a continuum of thoughts ranging from mild to severe concerning death, which include thoughts and feelings about death, hurting one’s self, or the “planning, conduct and outcome” of one’s own suicide. Suicidal ideation alone may lead to serious suicidal behaviours (4). According to clinicians, suicidal behaviours fall on a continuum starting from ideation to suicidal attempts to completion (5). Therefore, research on suicide ideation among adolescents warrants greater attention, given it potentially leads to suicide attempts and other health risk behaviours (6, 7). Notwithstanding, suicidal attempts have a strong effect on the immediate families, including the ripple effect on peers (8, 9).

As the suicide rate among adolescents continues to increase, suicidal ideation should be a crucial target in the systematic screening and identification of youngsters at risk of suicidal behaviour (10). Furthermore, suicide ideation research on adolescents is equally important due to the developmental transition that occurs which might instigate changes in family relationships, in the context of peers, and the increased use and consumption of alcohol and drugs (11).

Accordingly, the first step before implementing any suicide prevention and intervention programme for adolescents should be to firstly, identify the process of how they have become suicidal (12). Suicidal ideation among college students, for example, has been related to a variety of factors focusing mainly on the maladaptive characteristics of the suicidal individual and characteristics that may contribute to suicidal behaviours (13). These include a family history of suicidal ideation (14), low socioeconomic background (15), elevated levels of exposure to adverse life events (16), depression, hopelessness (17) and loneliness (18).

While previous literature has highlighted the risk factors associated with suicide like hopelessness and loneliness (18–20), limited attention has been attributed to the adaptive behaviours or prophylactic factors, such as the reason for living, which may prevent a person from considering or attempting suicide. Two studies by Linehan et al. (21) on concentration camp survivors described how personal beliefs regarding life and their expectations for the future kept them alive. Therefore, it is important to study the many reasons for living as well as suicide ideation when attempting to ascertain the reasons people engage in suicidal behaviour. Notably, the reasons for living provide a more positive and preventive aspect towards suicide (13).

The reasons for living and coping strategies can be protective factors for suicidal ideation. Protective factors are sources that can help to reduce the risk of suicide ideation or even suicide attempts. Moreover, the reasons for living constitute a protective factor defined as life-oriented beliefs and expectations that mitigate against committing suicide that can be measured using the Brief Reasons for Living for Adolescents study (BRFL-A) (21). Coping with suicidal ideation in this study includes the use of a variety of resources including the reasons for living.

Notably, our knowledge concerning how far general coping strategies and the reasons for living influence stress, anxiety and reduce suicidal thoughts remains limited. Past reviews and meta-analysis have highlighted that active forms of coping, such as problem-solving and support seeking; can have a beneficial effect in promoting mental health and reducing adjustment problems in response to controllable stressors (22, 23). Whereas other forms of coping, such as emotional regulation and distraction, have been used for relatively unalterable stressors (24). Further, those who mainly rely on avoidance or disengaging coping styles (e.g., resignation, escape, etc.) generally have poor adjustment and behavioural-emotional problems (24, 25).

In the manner in which young people cope with stressors depends on their ability to reduce their perceptions of stress and avoid the consequent effects on mental health (9, 23, 26). A study by Schotte and Clum (27) on the relationship between problem-solving skills, negative life stress, hopelessness and suicide ideation among college students found that poor problem solvers tend to have higher suicide ideation and are more hopeless in dealing with negatively life stress situations. Further, problem-solving and seeking support based on clinical samples (28) can conversely, have a protective influence against suicidal ideation. Promising results have also been observed in a limited sample population of urban adolescents at risk of dropping out of school employing behavioural intervention and coping-skills training programmes to reduce depressive symptoms and suicidal ideation (9, 29).

Therefore, this present study aims to identify the influence of the reasons for living and coping strategies in reducing suicidal thoughts and tendencies using multiple regression analysis, and how adolescents in Malaysia cope with their suicidal thoughts.

## Methods

### Participants and Research Design

This cross-sectional survey study was conducted among 176 adolescents aged between 13 and 19 years of age who volunteered to participate in this study, with the majority being Malays and Muslim. The inclusion criteria used for the selection of subjects included the age the of the person, between 13 and 19 years of age, their ability to read and write in the Malay or English language and their willingness to participate in the study. Subjects from special needs classes were excluded. The study was approved by the Universiti Kebangsaan Malaysia Research Ethics Committee (approval number NN-2018-060) and was supported by the Geran Galakan Penyelidikan (GGP-2017-059).

### Measures

#### Suicide Ideation Scale

The Suicide Ideation Scale (SIS) is a 10-item self-report scale developed by Rudd (30) to assess the severity or intensity of suicidal ideation among college students. The score of each item using the scale ranges from; 1 = “Never or none of the time” to 5 = “Always or a great many times”, which describes how often the respondent felt or behaved during the past year. The total score ranged from between 10 and 50. The SIS administered among college students showed a high level of internal consistency (Cronbach’s alpha = 0.86) as well as adequate item-total correlations (*r* = 0.45 to 0.74) (30). The concurrent validity was moderate with the Centre for Epidemiologic Studies - Depression scale (*r* = 0.55) and the Beck Hopelessness Scale (*r* = 0.49). In our study, both the English and Malay versions were administered. The reliability of SIS in this study was also found to be high (Cronbach’s alpha = 0.93) and the concurrent validity was low with the Depression Anxiety Stress Scale (DASS-21) (*r* = 0.17 to 0.27).

#### The Reasons for Living Inventory for Adolescents (RFL-A)

The Reasons for Living Inventory (RFL-A) was developed by Osman et al. (31) and consists of 32 items to measure five protective factor dimensions as preventive barriers of suicidal behaviour. A 6-point Likert scale is also used to determine the level of importance of each statement ranging from 1 = “not important at all” to 6 = “very important”. The dimensions in the RFL-A include Future Optimism, Suicide Related Concerns, Family Alliance, Peer Acceptance and, finally, the Self-Acceptance subscale. The reliability in the study was found to be high (α = 0.89) for the total score which ranged between 0.92 and 0.94 for the subscale score (31). The concurrent validity of the RFL-A was moderate with the Suicide Probability Scale (*r* = −0.60), Beck Hopelessness Scale (*r* = −0.65), and Depression subscale of the Brief Symptom Inventory (*r* = −0.48) (32, 33). Both the English and Malay versions of the RFL-A were administered in our study and yielded a high level of internal consistency value (α = 0.94), and the concurrent validity of RFL-A with Suicide Ideation Scale was found to be moderate (*r* = −0.45).

#### Jalowiec Coping Scale

Sixty items are used on the Jalowiec Coping Scale (JCS) to assess a wide range of coping strategies based on the Lazarus model of stress and coping (34). The JCS was initially designed to measure how people cope with various types of physical, emotional and social stressors and measures the use and effectiveness of 60 cognitive and behavioural coping strategies in a stressful situation. The items are rated on a 4-point response scale ranging from “never” (1), “sometimes” (2), “often” (3) to “almost always” (4). The coping strategies are grouped into eight coping dimensions: confrontative, evasive, optimistic, fatalistic, emotive, palliative, supportive, self-reliant. Jalowiec comprehensively evaluated the construct validity of the JCS and reported adequate internal consistency for three factors (Cronbach’s alpha 0.70–0.85). Both the English and Malay versions of JCS were administered to the adolescents in this study and resulted in a high internal consistency score (*r* = 0.93). The concurrent validity of JCS was found to be low to moderate with DASS-21 (*r* = 0.09 to 0.52).

#### Statistical analysis

All statistical analyses were performed using the Statistical Package for the Social Sciences (SPSS) version 22 and data were analysed using correlation and multiple regression analysis. Descriptive statistics were also used for the characteristics of the respondents in the study along with Pearson’s correlation to determine the relationships between the reasons for living, coping strategies and suicidal ideation, while multiple regression analysis was conducted to determine the contribution of reasons for living and coping strategies towards suicidal ideation.

## Results

A total of 176 adolescents, with ages, ranging between 13 and 19 years of age, participated in this study. The number of male participants outnumbered the female participants (males = 105, females = 71) and all participants were single. Further, the majority of participants were: Malay 149 (84.7%), Muslim 155 (88.1%), and most had attended school for six years or more, sitting for their UPSR (Primary School Achievement Test) except for five who had received no schooling (Table 1).

Table 2 shows the mean and standard deviation of all scales used in this study. The mean score for suicide ideation was 15.09 (SD = 7.92). The SIS score of 15 and above was categorised as those people with severe suicidal ideation as recommended by Rudd (30). There were 55 subjects (31.3%) with severe suicidal ideation in this study.

The relationship between suicidal ideation, reasons for living, and general coping skills showed that the reasons for living had a significant negative relationship with suicidal ideation (*r* = −0.323 to −0.475). Table 3 indicates that the greater the reasons adolescents had for living, the less their suicidal ideation. Furthermore, suicidal ideation among adolescents [in the present sample] decreased with increased concern for the future, moral objection, responsibility to friends and family, fear of suicide, fear of social disapproval and having more self-acceptance. Moreover, the general coping strategies represented by the Jalowiec Coping Scale had no significant relationship with suicide ideation except for a significant positive relationship with palliative coping (Table 3).

Further analysis using regression analysis with the stepwise method of entry showed that among the Reasons for Living subscales, only the Family Alliance subscale contributed significantly towards suicidal ideation. Four coping strategies contributed significantly towards suicidal ideation, namely; evasive, optimistic, emotive and palliative. Table 4 also shows that all factors contributed 31% towards the variance in suicidal ideation (Adj. R square = 0.311, *P* < .000).

## Discussion

While suicidal rates are increasing among young people, albeit the rates differ for each country (18, 19), the reasons for living constitute a key concept having an important cognitive factor (35) which might help to reduce suicidal ideation in adolescents. Although, having little reason to live might lead to suicidal thoughts and attempts (36). In this study, it was found that adolescents tended to have less suicidal thoughts as they had more reasons for living, greater fear of suicide, higher survival and coping beliefs, stronger moral objections, feel more responsibility towards family and friends, and with better self-acceptance. This finding is supported in prior studies (21, 37–39) in which the reasons for living were negatively associated with suicide ideation in younger adults. In general, the reasons for living may provide a sense of purpose and meaning that enables people to persevere and live through difficult circumstances (37, 40).

The interpretation of suicide very much depends on the cultural values (41), as does the interpretation of suicidal thoughts (42, 43). For instance, Malaysians come from different religious backgrounds and possess different moral and cultural values, which might strengthen their reasons for living, thereby helping to protect them against hopelessness, depression and suicide ideation. This is illustrated by the significant negative moderate association of all five subscales of RFL-A with suicide ideation in Table 3, where adolescents were shown to have strong survival and coping beliefs, strong responsibility to their family, fear of social disapproval and strong moral and religious objections which protect them from suicidal ideas. However, at the level of the regression model, only the Family Alliance subscale of RFL-A contributed significantly towards suicidal ideation, which indicated that the majority of adolescents in this study shared problems with their family.

In Malaysian culture, adolescents usually stay at home with their family even after reaching the age of 18 years of age, and many will remain living with their family until they are married. Whereas, those adolescents who move out from living at home with their family, are usually those individuals who obtain a job elsewhere or when they finally get married. The results also indicated that the adolescents in this study firmly hold onto and believe in family values and shared their problems with their family in times of need. This finding is also supported by the literature examining the reasons for living among African-Americans, which indicates that the ability to generate reasons for living is associated with strong cultural values, racial identity, religiosity and social support (44–46). Religion was also seen to influence the goals in a person’s life and contributing towards the meaning of life, which may have help to prevent suicidal thoughts and tendencies among the adolescents in this study. In the case of India, religion has been observed as a protective factor against suicide (47) and importantly, should be incorporated into suicide prevention programmes (48). Accordingly, culture, values and beliefs should be given due importance that can play a vital role in moderating suicide (13).

Furthermore, adolescents with a lower suicide ideation score also had a higher Peer Acceptance subscale score, which is consistent with the research conducted by Lamis (49), and Ellis and Jones (50). Adolescents are in great need of friends and a social network around them to understand them in times of stress and help to find a solution to their stressors (49, 51). Likewise, being in a period or, transition in life, social networks and relationships with friends can help to act as a buffer against certain social behaviours (49). Conversely, the lack of a strong social network can lead to depression and susceptibility to suicidal behaviours (49). In this case, the relationship between the reasons for living and suicidal ideation might not be directly linked, but instead are through the interaction with negative life stress, hopelessness, loneliness or depressed mood.

The findings in this study have shown that the reasons for living and general coping strategies are important protective factors to guard against suicidal ideation. These results were found to be consistent with other studies found in the literature where suicide ideation has been associated with life stress, hopelessness and depression. Furthermore, the reasons for living may help to protect adolescents during a vulnerable time or stage in their life to guard against suicidal thoughts.

Several studies have shown that the reasons for living are effective in protecting against suicide in people with major depression (21, 52) and the reasons were inversely related to suicide risk, suicidal ideation, and suicidal behaviour in nonclinical and clinical samples (13, 39, 50, 53). Also, suicide ideators had fewer reasons for living with lower coping mechanisms and strategies for managing life stressors and were more susceptible to suicidal behaviours (50). Interestingly, those individuals who contemplate suicide also consider the reasons for living (54, 55) and those who are able to generate an abundance of reasons for living may experience less suicidal intent (52, 56, 57), which indicates the importance of survival and coping beliefs of people.

In this study, the adolescents’ general coping strategies, as measured by the Jalowiec Coping Scale, showed a relationship between palliative coping strategy and suicidal ideation. The results indicated that one of the ways the adolescents were able to overcome suicidal thoughts was by keeping themselves busy (active) and working harder. Also, the multiple regression analysis in this study provided a much clearer picture of the way adolescents’ coped with their suicidal ideation; where suicide ideation was positively related to evasive thoughts and emotive coping and was negatively affected by optimistic and palliative coping strategies. Furthermore, the results also indicated that coping strategies might moderate the effects of stress in life and reduce the occurrence of suicidal behaviour. Notably, this may be explained by the diathesis-stress-hopelessness model of suicidal behaviour where problem-solving variables may moderate the effects of stress on hopelessness, which, in turn, affects the occurrence of suicidal ideation (27, 51, 58).

Other studies have also emphasised the role of cognitive appraisal in solving problems (59, 60) where high negative life stress and self-appraised ineffective problem solving are associated with higher levels of hopelessness and suicide ideation (61, 62). Therefore, it appears that stress and perceived ineffective problem solving independently increase the risk for hopelessness and suicide ideation (51). For example, a longitudinal study by Khurana and Romer (9) found that three types of coping strategy; problem-solving, support seeking, and emotional regulation, naturally adopted by male and female youth were protective in reducing the risk of suicidal ideation over a one-year follow-up period. These coping strategies were likewise found to influence suicidal ideation directly or mediate through perceived stress and depressive symptoms (9). This particular study was limited to a small number of Chinese subjects to investigate the cultural differences and religious effects on the reasons for living, coping and social support on suicide ideation. Therefore, since the reason for living, and especially family alliance and coping skills are related to suicide ideation, the potential intervention to help young people better manage stress and resist suicide as a solution to life’s problems should be implemented. Importantly, the support and acceptance from family and friends are critical during youth because the presence of supportive others in their lives not only provides them with a reason for living (52) but also provides them with an awareness and understanding of the experiences faced by others. Accordingly, effective, coping skill training programmes should also include the components of problemsolving, seeking support and emotional regulation to enhance the skills in managing many of life’s problems.

## Conclusion

This study examined the protective role associated with the reasons for living and coping strategies in helping to reduce suicidal ideation among young adolescents in Malaysia. A cross-sectional survey, also employing correlation and multiple regression analysis of 176 adolescents between 13 and 19 years of age was conducted. The findings of the study found that the reasons for living and palliative coping strategy correlated negatively with suicide ideation and that family alliance and palliative coping strategies are significant reasons for living that protect adolescents from suicidal thoughts. More importantly, that cultural and social values play an important role in protecting adolescents in Malaysia from suicidal behaviour.

## Figures and Tables

**Table 1:** Demographic characteristics of respondents

Variable	Category	Frequency	Percentage
Gender	Male	105	59.7
	Female	71	40.3
Age group	12–14 years	40	22.7
	15–16 years	55	31.3
	17–19 years	81	46.0
Education level	No formal schooling	5	2.8
	UPSR (six years of schooling)	36	20.5
	PMR (nine years of schooling)	59	33.5
	SPM (11 years of schooling)	56	31.8
	Certificate	10	5.7
	Diploma	9	5.1
	Degree	1	0.6
Race	Malay	149	84.7
	Chinese	2	1.1
	Indian	25	14.2
Religion	Islam	155	88.1
	Hindu	19	10.8
	Christian	2	1.1
Marital status	Single	176	100.0

**Table 2:** Mean and standard deviation for suicide ideation, reasons for living and coping

	Mean	SD
Suicide ideation	15.09	7.92
RFL-A future optimism	31.77	8.68
RFL-A suicide related concern	26.08	8.21
RFL-A family alliance	31.11	8.94
RFL-A peer acceptance	25.53	7.34
RFL-A self-acceptance	27.33	7.14
JCS confrontive	15.12	5.09
JCS evasive	17.87	6.73
JCS optimistic	14.65	4.94
JCS fatalistic	4.84	2.53
JCS emotive	6.04	2.67
JCS palliative	8.17	3.60
JCS supportant	7.74	2.70
JCS self-reliant	11.44	4.08

**Table 3:** Correlation matrix between suicidal ideation, reason for living and coping skills

	Suicide ideation
RFL-A future optimism	−0.421**
RFL-A suicide related concern	−0.323**
RFL-A family alliance	−0.475**
RFL-A peer acceptance	−0.421**
RFL-A self-acceptance	−0.439**
Reason for living total	−0.454**
JCS confrontive	−0.044
JCS evasive	0.139
JCS optimistic	−0.051
JCS fatalistic	0.119
JCS emotive	0.134
JCS palliative	0.153^*^
JCS supportive	−0.114
JCS self-reliant	0.008

** Correlation is significant at the 0.01 level (2-tailed).

**Table 4:** Regression analysis on the influence of reasons for living and coping on suicidal ideation

	B	Std. Error	Beta	AdjR^2^	*t*	*P*
(Constant)	1.936	0.140		0.311	13.807	0.000
RLF-A family alliance	−0.025	0.004	−0.454		−6.849	0.000
JCS evasive	0.028	0.007	0.393		3.924	0.000
JCS optimistic	−0.019	0.008	−0.197		−2.295	0.023
JCS emotive	0.033	0.014	0.179		2.410	0.017
JCS palliative	−0.025	0.013	−0.188		−2.010	0.046
